# Cell-to-cell signaling through light: just a ghost of chance?

**DOI:** 10.1186/1478-811X-11-87

**Published:** 2013-11-12

**Authors:** Ondřej Kučera, Michal Cifra

**Affiliations:** 1 Institute of Photonics and Electronics, Academy of Sciences of the Czech Republic, Prague, Czechia

## Abstract

Despite the large number of reports attributing the signaling between detached cell cultures to the electromagnetic phenomena, almost no report so far included a rigorous analysis of the possibility of such signaling.

In this paper, we examine the physical feasibility of the electromagnetic communication between cells, especially through light, with regard to the ambient noise illumination. We compare theoretically attainable parameters of communication with experimentally obtained data of the photon emission from cells without a specially pronounced ability of bioluminescence.

We show that the weak intensity of the emission together with an unfavorable signal-to-noise ratio, which is typical for natural conditions, represent an important obstacle to the signal detection by cells.

## Introduction

**Figure 1:**
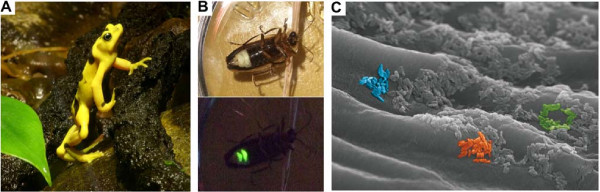
**Bio-communication through light.** Communication via visible part of electromagnetic spectrum is common in nature. Many organisms communicate by light reflected from their bodies, like strawberry poison frogs *Dendrobates pumilio***(A)**. Some organisms, for instance fireflies, are also able to generate light for the purpose of communication **(B)**. However, electromagnetic communication between single cells is a subject to speculations (an artist view **C**). (*Attribution: A - Davepape at en.wikipedia, B - Emmanuelm at en.wikipedia*).

Information transfer through signaling molecules is considered the basic principle of communication between cells. Electrical signaling also plays a role, but it is predominantly manifested as a convective electrical phenomena—*i.e.* the current of charged mass particles, usually ions—so it may be also understood as chemical signaling for the purpose of this article. With the exception of very local physiological events (involving, for instance, voltage-gated ion channels), the signaling through electromagnetic fields—for example light or radio waves—appears to be rare. In a broad sense, this is not true because many organisms communicate by changing the properties of daylight which is reflected from their bodies [1]; some organisms are even able to generate light for the purposes of communication [2]. Both of these examples are visible by the naked eye (see Figure 1). But could such a signaling—through light—take place between individual cells without a specially pronounced ability of bioluminescence? Could it be a general phenomenon in cell biology?

There are several good reasons to ask these questions. Firstly, well-established photo- and electro-chemical mechanisms make the cellular electromagnetic signaling possible in principle. Why shouldn’t organisms use available physical mechanisms to gain advantage in evolutionary fitness? Secondly, several experimental works attribute the observed effects to some kind of electromagnetic signaling [3]. Few hypotheses work with this concept, too. Therefore, experimental and theoretical motivation for studying the electromagnetic cell-to-cell signaling exists. Thirdly, if proved to be true, detailed knowledge of cellular electromagnetic signaling may help us learn more ways of how cell cultures can influence each other and how to prevent this interaction. This is vitally important for proper design of control experiments. Last but not least, understanding the possibilities of non-chemical signaling between cells is interesting by itself just from natural curiosity.

**Figure 2:**
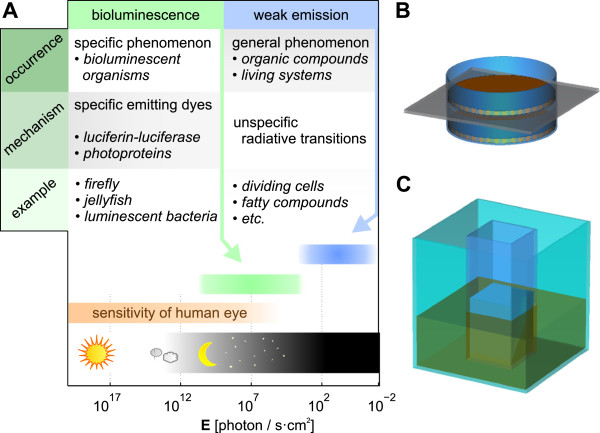
**Light emission phenomena in biological systems.** Typical intensity of bioluminescence is significantly higher than weak photon emission of unspecific auto-luminescence **(A)**. Typical setup for experiments dealing with cellular signaling through light is based on placing cell cultures to either Petri dishes, one put on the top of other **(B)**, or inserted cuvettes **(C)**.

In this paper, we discuss and analyze the possibilities of cell-to-cell electromagnetic signaling, mainly through light because there are no well established bio-mechanisms known which may serve for emission/perception in other parts of the electromagnetic spectrum. We review modern evidence of cellular signaling through light and summarize preconditions for such signaling. We show that an important limitation for signaling through light is given by the very low intensity of cells’ emissions (see Figure 2**A**). The results of our consideration indicate that information transfer using this weak light is possible but unfavorable signal-to-noise ratio which occurs under natural conditions represents an important obstacle to signal detection by cells.

## Electromagnetic signaling in biological context

### A brief review of experimental evidence of cell-to-cell electromagnetic signaling

There is a large number of experimental reports (over 400 papers published from 1920s onward) which describes signaling between chemically separated cell cultures proposed to be mediated by light or other electromagnetic radiation. Interested readers may find references in several recent reviews[3-5]. Although many of the older reports are considered highly controversial [6]—because they suffer from poor scientific standards and lack of critical evaluation— credible and further discussed experimental indications with forceful protocols also exist. These texts suggest that cells may utilize certain regions of the electromagnetic spectrum for signaling [7-12].

Present-day experiments which indicate electromagnetic cellular signaling are based on the evaluation of correlation of specified biological parameters between two cell cultures placed in detached neighboring cuvettes or Petri dishes (see Figure 2**B**, **C**). Observed correlations are then compared to control experiments. The distance between cell cultures in experiments varies most usually between the range of micrometers and millimeters; the maximum reported distance is 4 cm [10]. The results of these experiments show significant correlation of the growth rate, gene expression or other parameters between the detached cultures. The sealing of cuvettes or other measures for minimization of chemical interaction do not eliminate the interaction effect in any significant way. However, the effect disappears when a barrier, which is nontransparent in a certain frequency region, is placed between neighbor cuvettes or Petri dishes. This indicates that the interaction is dependent on the electromagnetic emission from the cell cultures.

Albrecht-Buehler disclosed that BHK cells were able to detect the orientation of the other layer of cells, which was separated from the first by a glass film [13]. It was possible to inhibit this effect by coating the glass film with a metal film. Coating with a silicon (transparent) film did not lead to inhibition. The author proposed infra-red radiation as the communication mechanism and further developed this idea in his later works [8]. Coupling between cell cultures in the optical band of the electromagnetic spectrum was also reported in the detailed study by Shen *et al.*[9]. Two cultures of neutrophils were separated chemically but not optically. One of these cultures was stimulated to undergo a respiratory burst while biochemical and biophysical parameters were recorded in both cultures. The response of the second culture resided in an increased level of chemiluminescence and generation of free radicals. Farhadi *et al.*[10] reported high correlation of the protein content, structural changes and NF- *κ*B (nuclear factor kappa-light-chain-enhancer of activated B cells) between two Cacco-2 cell cultures, which were again chemically separated and one of those was exposed to hydrogen peroxide. The correlation in the growth rate between separated populations of *Paramecia* was shown by Fels [11], who also proved a significant difference between separation by glass or quartz (which have different transparency in terms of frequency band). This fact can be interpreted as a dependence of signaling on the spectral transmission of the barrier. Electromagnetic signaling was also considered to be the mechanism of interaction between detached cell cultures (NIH3T3 and HMVECad cells) in the recent report by Rosi *et al.*[12].

There is no reason to question these results. However, we have to ask whether signaling through the electromagnetic field may provide a defensible explanation of the observed effects. The key uncertainty of the above-mentioned experiments is the degree of chemical separation. Although the authors took measures to minimize the possibility of chemical signaling between the observed cultures, no reported experiment, to our knowledge, shows absolute chemical separation including separated atmospheres, chemically identical surfaces in all controls, *etc*. It was proved that the sole closing of the containers with cells indeed reduces the possibility of chemical interaction but it is not the absolute protection. Therefore, the question is, whether the experiments show positive evidence of electromagnetic signaling, rather than lack of evidence for the chemical interaction. If the chemical interaction seems highly unlikely, what makes the electromagnetic interaction likely in an explicit sense? Does the effect of transparency of the barrier provide sufficient evidence?

### Preconditions for electromagnetic signaling

First of all, signaling and communication by any medium requires a mechanism modulating the information to be transferred into some physical property of the medium. This is then transmitted to the receiving structure where a mechanism of detection of this modulation has to exist. In order to transfer any information by means of electromagnetic waves, cells must be able to: 

1. generate electromagnetic radiation with specific properties, and

2. detect the electromagnetic field together with the ability to sense some particular properties of this field.

There is no doubt that many types of living cells emit electromagnetic waves with power significantly higher than that corresponding to solely thermal radiation. Non-thermal electromagnetic emission from cells is experimentally proved in the optical range. Auto-luminescence (sometimes termed as Ultra-weak Photon Emission), *i.e.* spontaneous emission of light, is a generally occuring phenomenon in metabolically active systems, even in those without specific enzymatic or protein machinery for bioluminescence [14-16]. While bioluminescence connected with specific luciferase or photoproteins has such intensity that is perceptible by the human eye, unspecific auto-luminescent phenomena have a very weak emission rate (see Figure 2**A**). Many organic substances also exhibit substantial photo-luminescence, *i.e.* re-emission of absorbed light. On the other hand, indubitable experimental evidence for non-thermal electromagnetic radiation at lower frequencies is missing [17].

Cells may also interact with incident radiation and sense the electromagnetic field. Well proved mechanisms of reception of the electromagnetic field include chains of photo-chemical reactions in the optical range, *e.g.*, those utilizing rhodopsin [18]. Besides specialized cells for detection of light [19], also other cells were shown to have the ability to sense electromagnetic waves using unidentified mechanisms. Albrecht-Buehler has, for instance, shown the ability of 3T3 cells to actively extend towards sources of infrared light, while the thermal effect of the irradiation was shown to be negligible [7]. Indisputable mechanisms of perception of the electromagnetic field on lower frequencies include thermal effects and, to a lesser extent, also resonant coupling to spin dependent chemical reactions [20] and resonant coupling to electrically polar structures [21]. Although many studies report significant biological effects of electromagnetic fields with frequency below the THz region, these results are generally hard to reproduce or contradictory to other results. Accordingly, only unspecific thermal effects of radio-frequency and microwave electromagnetic fields are widely accepted [22].

As a result, established mechanisms for the generation and perception of electromagnetic waves by cells exist only in the optical range.

### Questions concerning cellular signaling through light

As we have shown in previous paragraphs, signaling and bio-communication through light is possible in principle and it might also explain some unforeseen experimental results. So far, however, little is known about the specificity of the generation and reception processes of the light in non-specialized cells^a^. Concerning the cell-to-cell signaling through light, we may only speculate what is the size of the information that is to be transmitted, how is this information coded into the physical properties of the light or what is the coverage of the signal and how is it directed. And many other questions follow. How is the spectrum divided between different streams of information? How many cells/structures can we expect to use, and therefore share, the spectrum? How are interference and crosstalk (*i.e.* undesired effects between different channels/pathways) suppressed? How is the signaling secured from any possible eavesdropping of predators?

If we admit the possibility of the idea of signaling between cells through light, we must draw the conclusion that this signaling should be very sophisticated (like chemical signaling) to cope with the requirements arising from the above-mentioned questions. But even stricter requirements result from the presence of ambient background noise.

## Analysis and discussion

Non-specialized cells are hardly able to emit light with an intensity above the level of ambient illumination under natural conditions. Within a colony of standard model cells (without naked eye visible bioluminescence generated by specialized enzymes, *e.g.*, luciferases), somewhere between ones and thousands of photons in the visible part of the spectrum are emitted per 1 cm^2^ per second [23]. The background illumination may be as high as 10^15^ visible photons per 1 cm^2^ per second for direct sunlight and decreasing below 10^3^ visible photons per 1 cm^2^ per second only in very dark environments like caves. This means that the cells which are trying to receive the signal will be exposed more to the useless noise rather then to the signal carrying information. Therefore, the signaling between cells through light shall be possible only in real biological conditions if it can perform despite the presence of ambient noise. Only then it may have some specificity and produce a detectable effect. From the biological point of view, we need to distinguish between two possible regimes of the signaling.

The first regime does not allow for cooperative behavior between cells within the colony. This regime implies that the intensity of the total emission is just the summation of the individual contributions from all cells, each itself having the possibility of signaling. Therefore, a single cell should be able to emit roughly the same portion of the emission. This portion is supposed to represent the detectable signal. Therefore, a single cell would emit significantly less photons than the entire colony. Any comparison of the measured emissions to the number of cells is rare in literature. Ref. [15] reports 2 net counts per second of 7·10^7^ cells using a setup with estimated detection efficiency of 0.1*%* which leads to approximately 3·10^-5^ counts per cell per second. Ref. [24] reports 7 net counts per second from 1·10^5^ cells using a setup with estimated detection efficiency of 0.1*%* which leads to approximately 0.07 counts per cell per second. Thus, signaling by means of such a weak emission leads literally to the single photon regime. Single optical-photon communication under day-light conditions over large distances is not only theoretically possible but also experimentally attainable in artificial systems [25,26]. However, this goal can be achieved only by spatial, time and spectral filtering and contrast enhancement. It means that both the transmitter and the receiver are spatially aligned with very high precision and only a direct beam of the signal from the transmitter is allowed to reach the receiver’s detector. Since propagation of light is very directional in atmospheric conditions, any usage of such spatial filtering leads to a dramatic improvement of the signal-to-noise ratio (ratio between power of signal, *S*, and noise, *N*, in Watts). Although the distance between the cells in reported biological experiments is only a few centimeters in maximum compared to kilometers in technical single photon experiments, the reduction of the length is offset by increased intricacy of the optical path (many interfaces, dispersion, *etc*). It is still uncertain whether single cells are capable of performing any kind of spatial filtering, let alone under such complicated optical circumstances. It is known that only eyes, *i.e.*, organs composed of large number of cells, are able to perform spatial filtering. We can also speculate whether cells can somehow exploit entanglement, angular momentum of photons, or whether they are able to perform modulation of the wave packet of a single photon. However, we still encounter very low probability of detection of modulated signaling photons by the detector cell because the emission level from emitting cells is extremely weak and there is probably no mechanism which can be used for precise spatial alignment between source and detector cells.

The second regime of signaling illustrates the eventuality of cooperative signaling. It this case, all cells would cooperate in order to emit detectable signals. The total emission would be either the summation of the coordinated weak contributions or it would be produced by just a few cells from the colony which would communicate with the rest of their neighboring cells by chemical signaling. Regardless of the plausibility of these two options, the total measured intensity of emissions should represent an individual detectable signal. Signaling by means of overall emissions would be far from the single photon regime, yet so far from standard signaling with the intensity of the signal higher than the intensity of the noisy background illumination in the majority of environments. In reality, optical noise may vary between direct Sun-light exposure (about 10^21^ photons/second/square meter) and very dark circumstances (few photons/second/square meter) in light-tight chambers, deep oceans or caves. Unfortunately, there is no systematic work dealing specifically with the influence of background illumination on the interaction effect. Such investigation might reveal the threshold level of the signaling and help to experimentally estimate noise limitations as well as other parameters of the communication. The majority of reports only state that the experiment was done under moderate indoor illumination or gloom. Since no photo-metric data were provided in those reports, we may only theoretically analyze whether the signaling can take place under similar conditions. Since the wavelength or bandwidth of hypothetical signaling is not known, we will use a general approach of the communication theory and combine it with the established bio-physical data.

### Communication in the presence of noise

The limitations of information transmission over a noisy channel are summarized in the famous Shannon’s theorem [27], a mathematical formula describing the capability of a system, either artificial or natural, to detect any kind of signal under specified noise conditions. Shannon showed that the maximum theoretical capacity, *C*, of a communication channel (in bits per second) is proportional to the channel’s bandwidth, *B* (in Hertzs), and defined the logarithm of the signal-to-noise ratio as $C=B\underset{2}{\log}\left(1+\frac{S}{N}\right)$. This equation states the minimum bandwidth required for communication with rate *C* under conditions of the given signal-to-noise ratio. Strictly speaking, we do not know the size of the information nor the speed required for cellular signaling through light, but we may estimate the plausible range of values of these parameters from the physical feasibility of *B* and *S*/*N*.

Concerning the feasible bandwidth, *B*, we may expect that its range should be covered by a single physical mechanism which involves electromagnetic waves. The first reason is that cooperative involvement of different mechanisms—for instance vibrations of polar molecules coordinated with photon emissions—seems unlikely^b^. The second reason resides in the fact that the conditions for propagation of electromagnetic waves with extremely different wavelengths may drastically differ. The evolutionary approach would lead cells to occupy those frequencies which naturally have the lowest background noise, *i.e.* long wavelength radio waves, micro-waves and short ultra-violet wavelengths (UVB, UVC)^c^. No well described mechanism of reception in cells is, however, known for radio waves. Longer exposure to short UV wavelengths is lethal for living systems. Thermal emission maximum of living systems lies within the infrared range. Therefore, this band should not have any special preference for communication, too, unless cells were able to modulate their thermal emission. So, cells may probably not take the advantage of electromagnetic “silence” on some frequencies. Thus, the maximal bandwidth we may take into consideration is difficult to estimate. However, we may expect that its upper limit will not substantially exceed the frequency extent of the human eye because frequencies higher than visible light have a potentially dangerous impact on living systems. Therefore we use the maximum bandwidth of 800 THz in the following considerations, which is roughly twice the range of the visible spectrum.

The number of principles may be, and indeed was, proposed to show how cells could achieve a more favorable signal-to-noise ratio^d^. The straightforward solution resides in the suppression of the power of the present noise by controlling the environment it lies in. For instance, by the development of special propagation pathways. This might be possible for large organisms, but unrealistic for single cells. Substantial noise suppression might also be achieved by the spatial filtering, *i.e.* allowing light only from a defined direction to reach the detector. Eyes are capable of performing spatial filtering, but single cells would need extensive machinery to be able to do so as well. Cells would either have to know the positions of other cells or have to selectively scan light from all directions. Both options appear improbable. Nevertheless, signal processing still enables some level of noise suppression if some properties of the specific noise and the signal are known. As we have learned from the signal theory —in general words—it is possible to reconstruct the useful periodical signal hidden in noise by averaging several realizations of the received signal. This can be done because the noise is not correlated between its realizations while the useful signal is. However, this technique has limited efficiency (because many realizations of the signal are needed) and it requires a high level of synchronization between the source and the detector. Problems with synchronization and filtering may be overcome by a phase-lock loop. Other possibilities of noise reduction reside in narrow band filtering. These techniques, however, have low efficiency for very low values of the signal-to-noise ratio. Other possibilities of detection, like intensity thresholding or stochastic resonance [28,29], can’t be excluded but are very likely limited to special conditions because natural variance of ambient noise intensity is, compared to the emission from cells, too high. Stochastic resonance can increase *S*/*N* by few orders of magnitudes only at very specific intensity levels of noise [30]. Some authors also proposed that ambient noise may pump the energy into specific systems within the cells and this energy may accumulate at a distinct frequency. The frequency conversion is common to non-linear systems and the process of generation of higher harmonics is common to some bio-molecules (it is widely used for imaging, see *e.g.*[31,32]). Any kind of energy condensation would manifest itself as an increased intensity of emissions at some distinct frequency range with a more favorable signal-to-noise ratio. However, conversion to a range with better *S*/*N* has not been experimentally reported to our knowledge^e^. Therefore, we may conclude that the proposed concepts suggesting the possibility of signal-to-noise ratio enhancement by cells are probably not able to provide a general plausible explanation.

**Figure 3:**
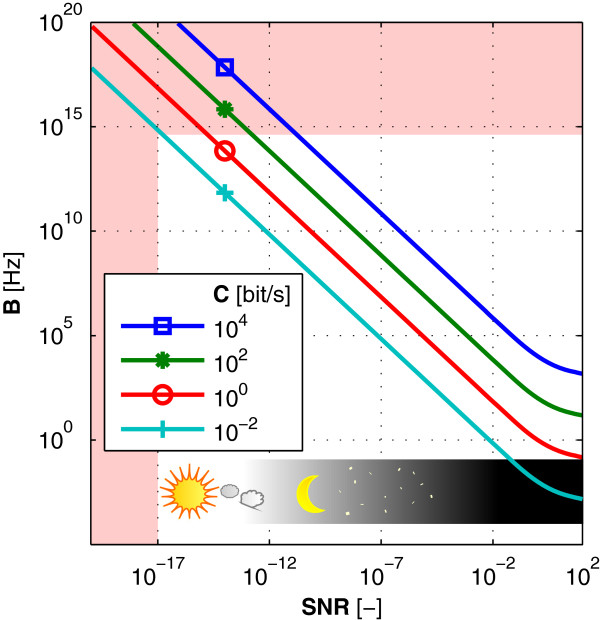
**Communication in presence of noise.** Principle limitations of communication in presence of noise are given by Shannon’s theorem, which shows the relation between bandwidth, signal-to-noise ratio and theoretical capacity of a communication channel. Curves displayed in the graph show comparison of frequency bandwidths required for communication under given noise circumstances. Each line corresponds to certain speed of information transfer. Signal-to-noise ratio was calculated for average emission of 100 photons/second.

Outcomes of these considerations are reflected in Figure 3, where the reddish areas limit the space of Shannon’s theorem to realistic values of parameters. Ranges of bandwidth and the signal-to-noise ratio then restrict the maximum theoretical amount of information that can be sent between cells. For extremely adverse noise conditions of direct Sun-light illumination (*S*/*N*=10^-17^) even the very wide frequency spectrum *B*=10^15^ Hz is not broad enough to send just the *Hello world!* message in reasonable time, nor more complicated information at all (note that resulting maximal speed of communication is below 10^-2^ bits/second). For *S*/*N* below 10^-12^, *i.e.*, from a slightly darker towards very dark conditions, the breadth of the required spectrum is comparable to the width of the emission spectra of bio-molecules and the information content per second is sufficient for simple signaling. Therefore, cellular signaling through light under the reported conditions could be possible. Unfortunately, the drastically limited number of emitted photons leads to a very restricted frequency bandwidth. Thus, we can conclude that even though the bio-communication with a highly unfavorable signal-to-noise ratio is possible in principle, it cannot work with such a limited number of photons. This conclusion may be supported by experimental data from photosensitive cells exposed to few-photon coherent signals. Experiments with highly photosensitive rod cells show that detectable responses occur at the minimum of a few tens of photons from a coherent source [33]. If non-specialized cells had the same sensitivity, the signaling through light (with its intensity similar to that routinely measured in cell photo-emission experiments) would be possible only in almost completely dark environments.

In conclusion, we can state that cell-to-cell signaling through light with reported intensities is physically possible only in extremely dark environments where the signal-to-noise ratio is relatively high and the necessary bandwidth is rather narrow. Other possibilities reside in the modulation of the properties of individual photons. However, the existence of mechanisms in biological systems for this purpose is not known. It is also not clear whether the available degrees of freedom of the properties of photons can possibly provide any information transfer without time/spatial filtering and synchronization.

### Future directions

So, what do the reports mentioned in the introductory review tell us? Even though the above-discussed limitations support the conclusion that signaling through light may probably not provide credible explanation of the reported effects on the basis of current knowledge, the involvement of light in cell-to-cell signaling cannot be completely disproved. Whether is it light, sound, or volatile compounds, the exact mechanism of interaction between cell cultures mentioned in the introductory section must be identified.

In fact, almost all reports mentioned in the introductory review were already driven by the hypothesis that cell signaling is mediated by light, so their authors have not performed measures to undoubtedly exclude other mediators of signaling. Therefore, more sophisticated bio-communication experiments with precise separation of cell cultures are necessary. The design of such an experiment should include isolated atmospheres, identical surfaces of cuvettes and vibration isolation. The barrier with modified spectral transparency which is in fact the only indicator of light involvement in cell signaling, should not come into direct contact with cells in order to prevent any changes in the chemical and/or surface properties of the environment. This means that all cells in the experiment must be in contact with exactly the same surface in order to prevent any physical and chemical effects caused by different surfaces. All relevant parameters like illumination, temperature and chemical properties of the media must be exactly the same for all samples. Needless to say, thorough control experiments are vitally important.

In our opinion, an alternative explanation of the observed effects should be sought. While other mechanisms, for instance chemical (volatile compounds) or acoustical (cellular vibrations) pathways, may provide an alternative explanation of the observed effects in certain experimental setups, none of the suggested mechanisms are able to provide a watertight explanation of the observed effects in all the setups. The feasibility of any proposed mechanism would deserve deeper analysis which goes beyond the scope of this paper. Although we advocate that the effects are hardly explicable by light (especially in experimental setups where the ambient light intensity is obviously high), we hold the view that bio-signaling experiments show us the fascinating ability of cells to detect information. Still, not enough is known about the auto-luminescence properties of living systems. However, a detailed analysis of the properties of photo-emission from cells may bring important data not only for the assessment of hypothetical signaling but also for non-invasive label-free diagnostic purposes.

## Conclusion

The conclusion we offer is that cellular signaling through light, if it exists as a general phenomenon, employs principles and techniques which are not expected to exist in living cells based on the currently accepted understanding of biophysical processes. So far, it seems that cellular signaling through light (on the level of single cells) is either 

1. an example of Foster’s “mechanisms paradox, *i.e.* the absence of established mechanisms by which electromagnetic fields at levels found in ordinary environments could produce observable changes in biological systems” [34], or

2. is not accomplishable under natural conditions and the reported experiments mentioned in the introductory section should be attributed to another phenomenon.

In this paper, we have shown how the idea of cellular signaling through light as a general phenomenon has fundamental limitations given by the ambient noise, which has, in the majority of circumstances, a much higher intensity than the endogenous cellular signal. Cells indeed emit light. However, as a by-product of bio-chemical processes, it has a very weak intensity. This light may bear information about its generation processes, but the detection of such a weak signal would require such mechanisms that would be very difficult, if not outright impossible, to find in biological systems. We may conclude that the signaling between cells which employ electromagnetic waves is possible in principle, but it is very likely limited to very special circumstances or it would require physical mechanisms which are inapplicable in biology or yet unknown.

## Endnotes

^a^ In this context we use term “specialized cell” to refer to cells which have highly pronounced ability of bioluminescence. The reason for this exclusion is that we are analyzing the cell-to-cell signaling through light as a general phenomenon present in the majority of cells.

^b^ Non-linear interactions; however, may broaden the bandwidth.

^c^ Under day light conditions, it may be dark only at very short (230 nm - 280 nm) UV wavelengths [35]. Historical and very controversial works of Gurwitsch claim that the photon emission from cells lies in the UV region (190 nm - 250 nm) [36], but there is just little modern evidence even for longer UV wavelengths (280 nm - 390 nm) in biological photon emission [37]. Mainly, there are no generally accepted mechanisms for generation of UV emission via endogenous chemical excitation in biological systems.

^d^ Note that many of proposed principles are tied up with the bandwidth.

^e^ Required intensities of the excitation laser light at long wavelengths were, in above mentioned reports [31,32], about 50 MW / cm^2^ which is several orders of magnitudes more than those provided under daylight. We do not know the spectra of photon emission (combined with fluorescence, phosphorescence and light induced chemiluminescence) under exposition by natural daylight because it is not possible to distinguish a photon from a technical source or the Sun from the photon emitted from biological system by a simple measurement. From the auto-fluorescence of tissue [38] we may estimate that the intensity of re-emitted photons is about 10^2^ to 10^6^ weaker than the intensity of exciting light.

## Competing interests

The authors declare that they have no competing interests.

## Authors’ contributions

OK conceived the study, performed the analysis and wrote the paper. MC supervised the writing and critically revised the manuscript. Both authors read and approved the final manuscript.
